# Bioinformatics gene analysis of potential biomarkers and therapeutic targets of osteoarthritis associated myelodysplastic syndrome

**DOI:** 10.3389/fgene.2022.1040438

**Published:** 2023-03-09

**Authors:** Peicheng Xin, Ming Li, Jing Dong, Hongbo Zhu, Jie Li

**Affiliations:** ^1^ Department of Orthopedics, The Second Affiliated Hospital of Shandong First Medical University, Taian, Shandong, China; ^2^ Department of Hematology, The Second Affiliated Hospital of Shandong First Medical University, Taian, Shandong, China

**Keywords:** myelodysplastic syndrome, osteoarthritis, bioinformatics, hub gene, signaling pathway

## Abstract

**Objective:** Osteoarthritis (OA) and Myelodysplastic syndrome (MDS) are diseases caused by the same immune disorder with unclear etiology and many similarities in clinical manifestations; however, the specific mechanisms between osteoarthritis and myelodysplastic syndrome are unclear.

**Methods:** The expression profile microarrays of osteoarthritis and myelodysplastic syndrome were searched in the GEO database, the intersection of their differential genes was taken, Venn diagrams were constructed to find common pathogenic genes, bioinformatics analysis signaling pathway analysis was performed on the obtained genes, and protein-protein interaction networks were constructed to find hub genes in order to establish diagnostic models for each disease and explore the immune infiltration of hub genes.

**Results:** 52 co-pathogenic genes were screened for association with immune regulation, immune response, and inflammation. The mean area under the receiver operating characteristic (ROC) for all 10 genes used for co-causal diagnosis ranged from 0.71–0.81. Immune cell infiltration analysis in the myelodysplastic syndrome subgroup showed that the relative numbers of Macrophages M1, B cells memory, and T cells CD4 memory resting in the myelodysplastic syndrome group were significantly different from the normal group, however, in the osteoarthritis subgroup the relative numbers of Mast cells resting in the osteoarthritis subgroup was significantly different from the normal group.

**Conclusion:** There are common pathogenic genes in osteoarthritis and myelodysplastic syndrome, which in turn mediate differential alterations in related signaling pathways and immune cells, affecting the high prevalence of osteoarthritis and myelodysplastic syndrome and the two disease phenomena.

## Introduction

Osteoarthritis (OA) is a chronic disease characterized by joint destruction, osteophytes and degenerative lesions of articular cartilage, while the pathogenesis of osteoarthritis is still unclear, and according to relevant studies, osteoarthritis is the result of a combination of factors (Watts et al., 2022; Yang et al., 2022). With the continuous development of modern medicine, it has been found that the immune system plays an important role in the OA. Immune cell infiltration mediates autoimmune response to osteoarthritis, inducing the secretion of chemokines, pro-inflammatory cytokines and proteases, which in turn disrupt the immune balance to accelerate cartilage erosion (Li et al., 2021b; Hao et al., 2021; Franco-Trepat et al., 2022). A larger scale epidemiological data study confirmed that compared with the matched control group, patients with autoimmune diseases have an increased risk of developing Myelodysplastic syndrome (MDS). Osteoarthritis is not caused by mechanical trauma, but caused by immune imbalance (Dalamaga et al., 2002).

Myelodysplastic syndrome (MDS) is a heterogeneous group of clonal diseases originating from hematopoietic myeloid-directed stem cells or pluripotent stem cells, and about 10% of patients with MDS may develop secondary to autoimmune diseases (Dong et al., 2022). It has been shown that some patients with MDS have a higher probability of secondary autoimmune disease than their natural incidence, while others have no clinical manifestations of immune disease but may have abnormal immunologic parameters, providing further evidence of the association between MDS and autoimmune disease (Wang et al., 2022). The pathogenesis of MDS is related to autoimmunity against hematopoietic stem cells, which may lead to abnormal clonal development of hematopoietic stem progenitor cells due to autoimmune reactions triggered by some specific immune stimuli, such as abnormal T cell response to antigen or abnormal T-B cell interaction, which can cause autoimmune diseases in other tissues and organs of the body along with hematopoietic stem cell destruction (Fu et al., 2019; Moskorz et al., 2021; Votavova et al., 2021).

The use of platelet-rich plasma (PRP) in the treatment of patients with OA complicated by MDS has had poor outcomes, and reports suggest that possibly MDS affects trilineage bone marrow dysplasia and influences the prognostic outcome (Li et al., 2022). However, there are few reports on the relational nature of the two. Therefore, it is important to clarify the potential connection between the two for the prevention and treatment of OA and MDS. Bioinformatics is an interdisciplinary discipline based on electronic information technology to conduct relevant research in the field of biomedicine, which can search for potential patterns between diseases from the perspective of gene molecules. Therefore, this study was conducted to screen and identify the common disease genes of OA and MDS based on bioinformatics, and to provide theoretical support for the prevention and treatment of the combined disease of OA and MDS.

## Materials and methods

### Data collection

Gene chips of MDS and OA patients were queried from the GEO public database and the MDS gene chip GSE19429 was downloaded. The GSE19429 chip contained 183 samples from MDS patients and 17 samples from healthy individuals. Also downloaded OA gene chip GSE55235 containing 10 samples from OA patients and 10 samples from healthy individuals, where see Table 1. Normalized the expression matrix and analyzed the differential genes between patients and controls using the limma package, where the screening conditions were *p* < 0.05 and |logFC|>0.5, to obtain the DEG of MDS versus OA, Venn diagram to determine the co-expression of differential genes. Using the “ggplot2” and “pheatmap” R packages, gene expression in the normal and MDS groups could be visualized.

**TABLE 1:** Sequencing chip data information.

Dataset id	Contributors	Disease type	Platform	Sample size (cases)	
				Patient group	Healthy controls
GSE19429^(Pellagatti et al., 2010)^	Pellagatti A, et al.	MDS	GPL570	183	17
GSE55235^(Woetzel et al., 2014)^	Woetzel D, et al.	OA	GPL96	10	10

### Functional enrichment of DEG

The GO/KEGG Analysis tool is a functional annotation tool based on the clusterprofiler package in R language (https:/hiplot.com.cn/advance/clusterprofiler-go-kegg) (Wu et al., 2021), which can independently select the latest GO and KEGG libraries for functional annotation. The conventional gene function enrichment analysis includes gene ontology (GO) and signaling pathwaykyoto encyclopedia of genes and genomes (KEGG), where GO enrichment analysis can be divided into molecular function (MF), GO enrichment analysis can roughly compare and classify DEGs to understand their biological properties, while KEGG analysis helps to understand the position and function of genes in the overall network of signaling pathways.

### Protein-protein interaction (PPI) analysis

The PPI network was constructed using the STRING database (Szklarczyk et al., 2019) by incorporating differential gene-encoded proteins and their directly related proteins, setting the interaction score >0.4 as the screening criterion, and then performing network topology analysis, and using the “cytoHubba” plug-in included in Cytoscape software. The top 10 genes with the highest scores were mined using the MNC calculation method in Cytoscape software, and the PPI modules were filtered by using the “MCODE.”

### cBioPortal database analysis

The cBioPortal (Brunner et al., 2022) (http://cbioportal.org) is an open web resource for querying, analyzing and visualizing multidimensional cancer genomic data from multiple databases, selecting MDS-related datasets and applying the Oncoprint module to analyze Hub gene variants.

### Validation of diagnostic markers

The value of the obtained genes as diagnostic markers was validated in two independent datasets, GSE19429 and GSE55235, by plotting receiver operating characteristic (ROC) curves for the characteristic genes obtained by taking the intersection set above and evaluating their diagnostic value with a threshold value of *p* < 0.05 to be determined. The Area Under Curve (AUC) was calculated, and the AUC was taken to be in the range of 0–1. The larger the AUC, the better the predictive performance.

### Immune-infiltration analysis

The expression matrices of immune cell subtypes were deconvoluted using the CIBERSORT to calculate the relative proportions of 22 immune cell types (Lu et al., 2021), and a *p* value was obtained for each sample. The barplot function of the “graphics” package of R was used to plot the histogram of the composition ratio of each immune cell in two groups of samples; The “vioplot” package of R was used to correlate the immune cells of patients. “Vioplot” package in R language was used to compare the ratio of immune cells in the healthy control group and patient groups and to draw violin plots, we analyzed the difference of population immune cells through the rank sum test, and took the comparison of *p* < 0.05 as a meaningful judgment.

## Results

### Common disease genes in osteoarthritis and myelodysplastic syndromes

**FIGURE 1:**
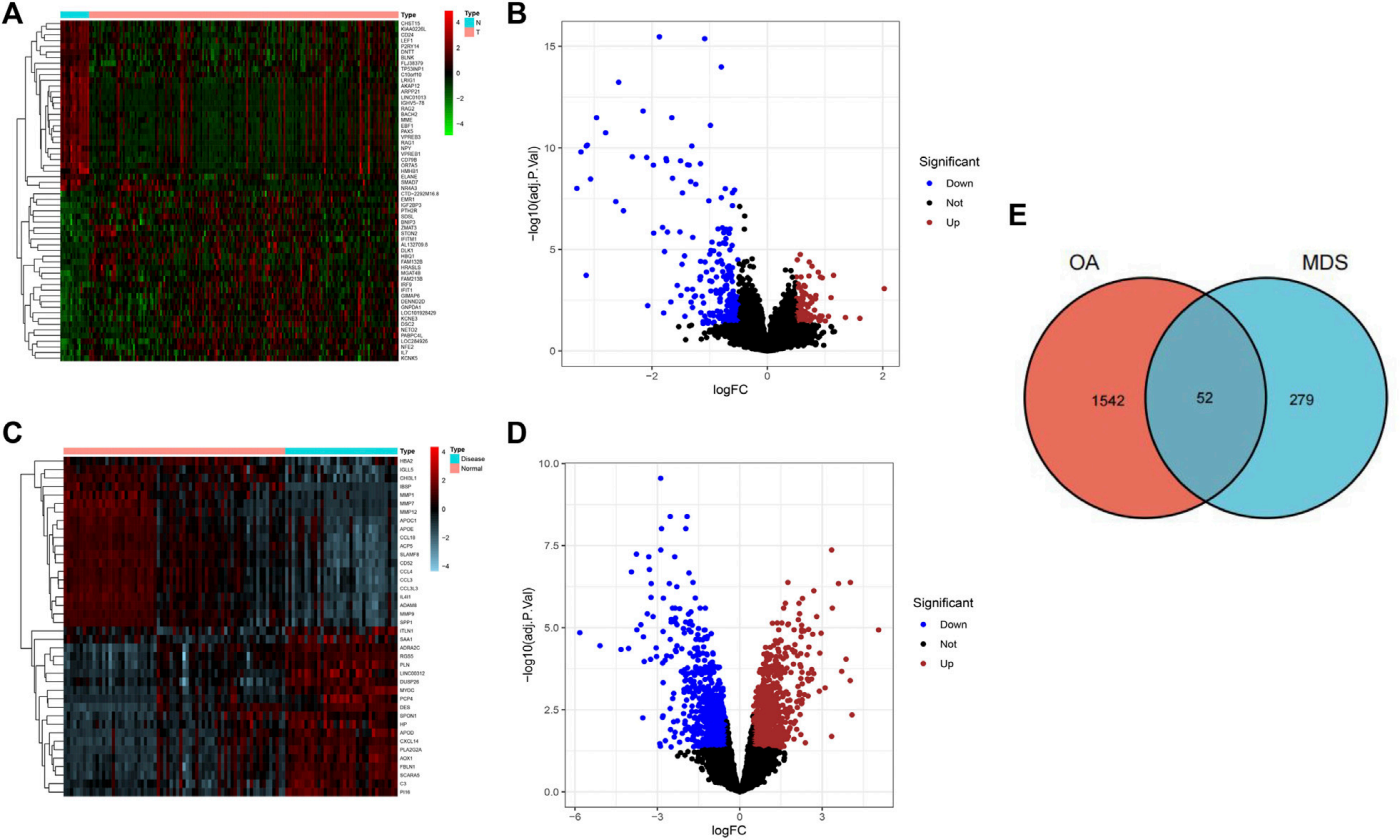
Screening results of DGEs for osteoarthritis and myelodysplastic syndromes. **(A)** Heat map showing the top 50 OA-associated DEGs.**(B)** Volcano plot showing 331 OA-associated DEGs. **(C)** Heatmap showing the top 50 MDS-associated DEGs **(D)** Volcano plot showing 331 osteoarthritis-associated DEGs. **(E)** Wayne diagram indicating common disease genes.

A total of 331 OA-related DEGs (106 upregulated genes and 225 downregulated genes) and 1594 MDS-related DEGs (840 upregulated genes and 754 downregulated genes) were obtained from the analysis of the osteoarthritis gene chip GSE55235 and the myelodysplastic syndrome gene chip GSE19429, as shown in Figures 1A–D. The DEGs obtained were intersected using the R language package “VennDiagram,” and 52 common disease genes were obtained, as shown in Figure 1E.

### Co-expression of differential genes for functional enrichment

**FIGURE 2:**
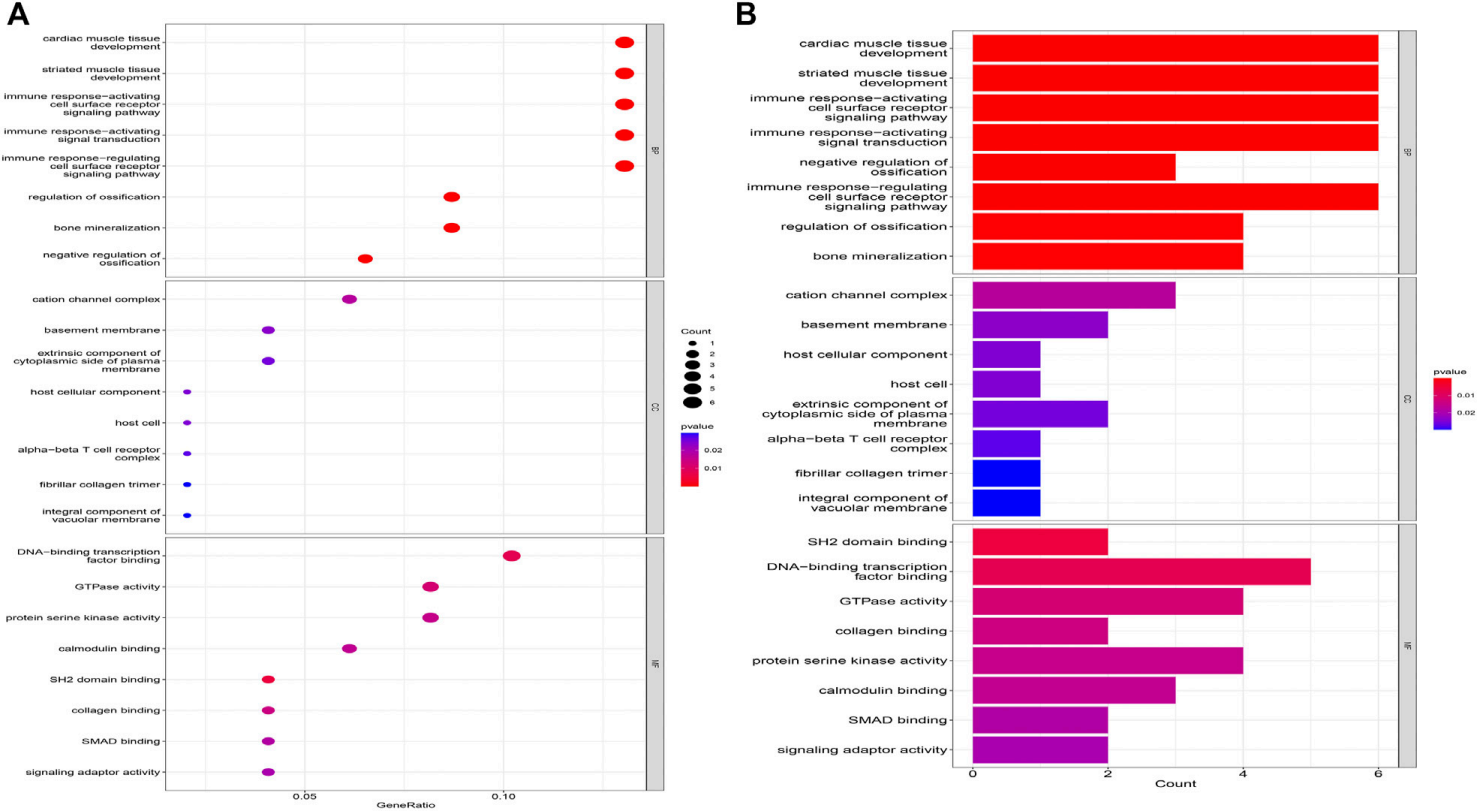
Results of GO analysis. **(A)** Bubble diagram; **(B)** bar graph.

**FIGURE 3:**
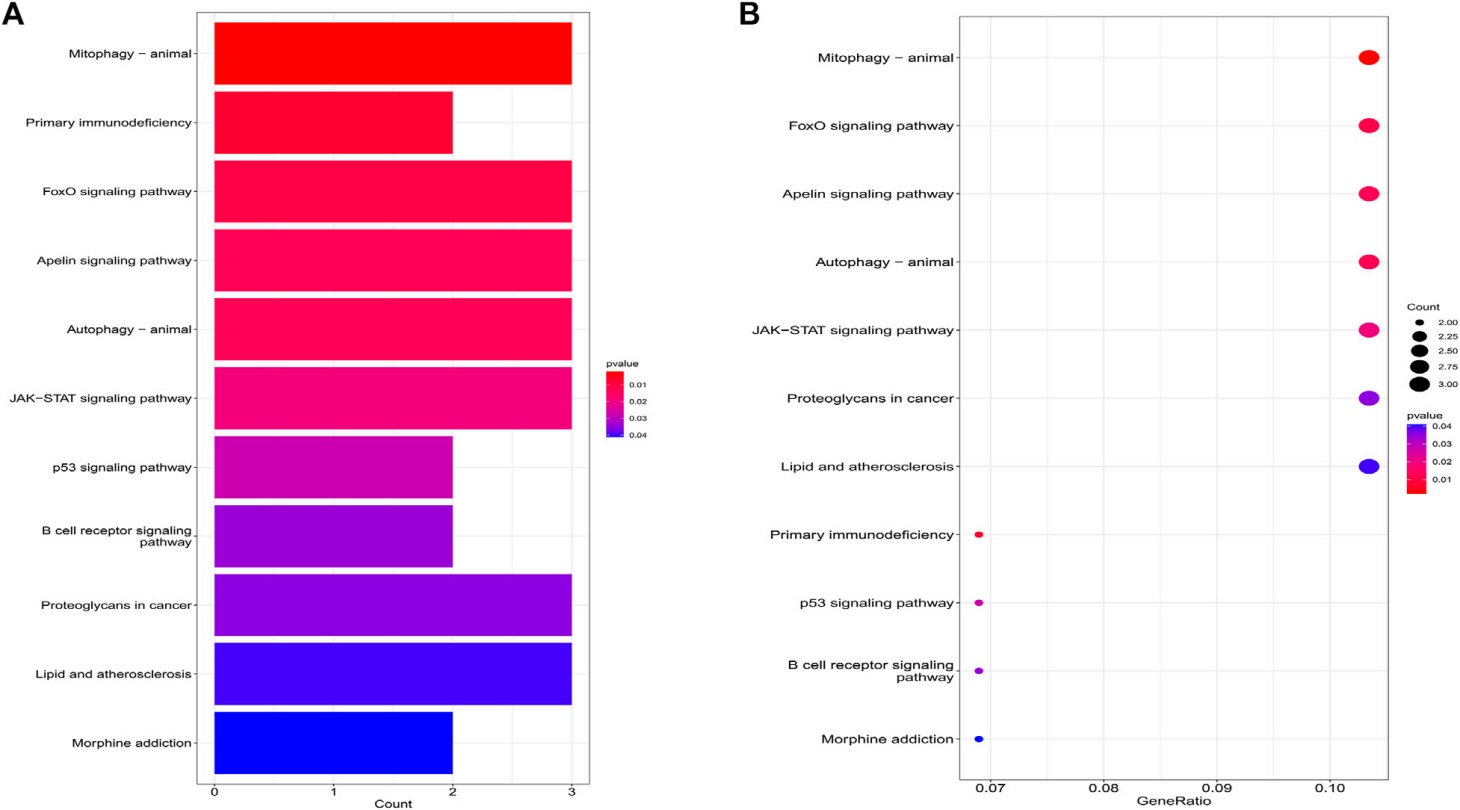
Results of KEGG analysis. **(A)** Bubble diagram; **(B)** bar graph.

The biological processes of co-expressed differential genes in osteoarthritis and myelodysplastic syndromes are focused on cardiac muscle tissue development, striated muscle tissue development, immune response-activating cell surface Cellular components are clustered in the cation channel complex, basement membrane and host cellular component. The molecular function component is focused on SH2 domain binding, DNA-binding transcription factor binding, and GTPase activity (Figures 2A, B); KEGG is concentrated in Primary immunodeficiency, FOX signaling pathway and B cell receptor signaling pathway. These pathways reveal a strong link between immune cells and immune activation pathways and the development of both diseases. Activation pathways are closely related to the development of both diseases (Figures 3A, B).

### PPI network and modularity analysis

**FIGURE 4:**
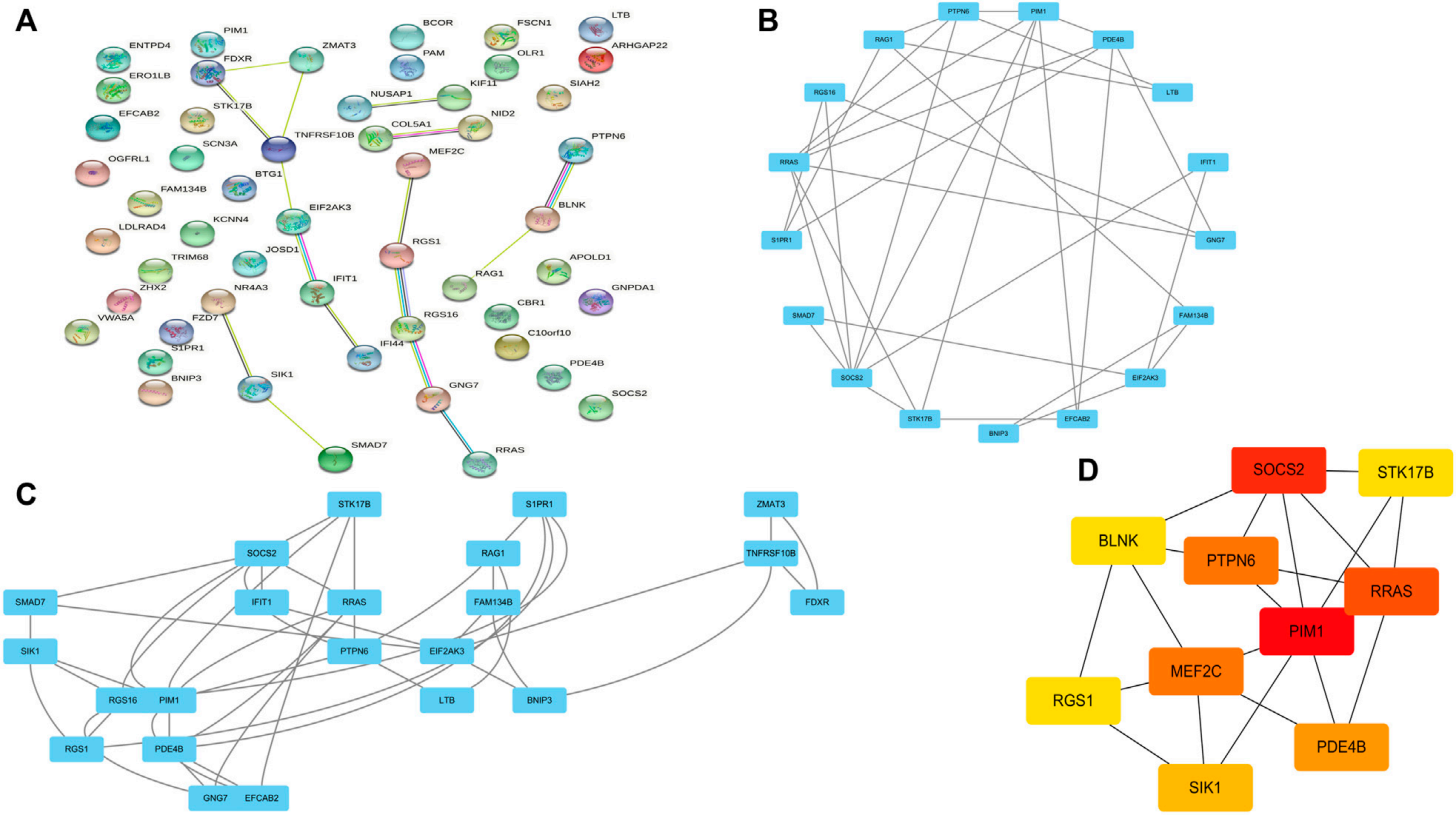
PPI network construction and functional module analysis. **(A)** PPI network; **(B)** Module 1; **(C)** Module 2; **(D)** 10 hub genes of PPI network.

Co-DEGs were analyzed using the String database (Figure 4A). Then, cytoscape to construct a PPI network containing 52 nodes and 101 edges. Subsequently, cluster analysis was performed using the Cytoscape plugin MCODE, and two functional modules were identified from the entire network (Figures 4B, C). We assessed the degree of core and intermediate in the PPI network and screened 10 genes to be screened as key diagnostic genes for osteoarthritis and myelodysplastic syndromes, top 10 hub genes were BLNK, SOCS2, SIK1, RGS1, STK17B, MEF2C, PDE4B, PIM1, RRASE, and PTPN6 (Figure 4D). Next we wanted to further analyze the function of the genes, so we performed GSEA analysis regarding their variation in MDS with OA, GSEA enrichment analysis showed activity in Immune_system_development, negative_regulation_of_response_to_stimulus, and anatomical_structure_formation_involved_in_morphogenesis play a decisive role in the process, and the results are shown in the Supplementary Figure S1.

### Hub co-expression gene screening and validation

**FIGURE 5:**
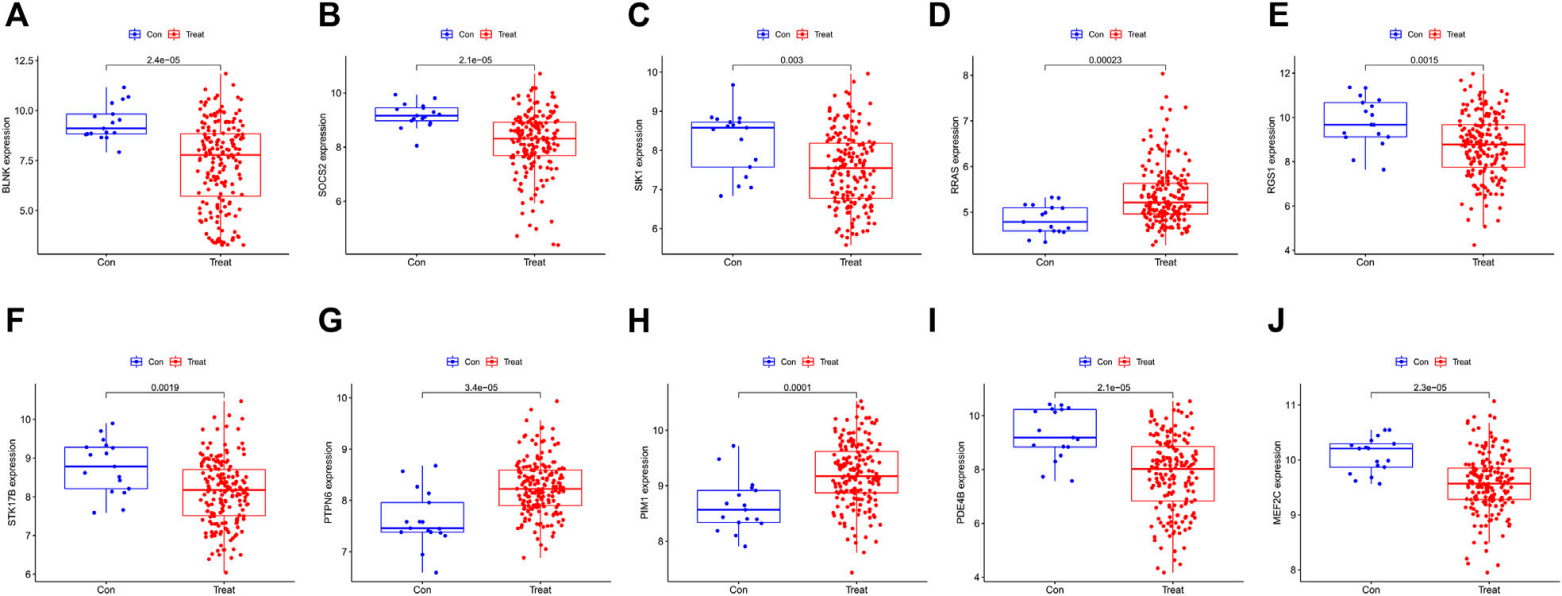
Box plots showing the differences in the expression of hub genes in MDS group.

**FIGURE 6:**
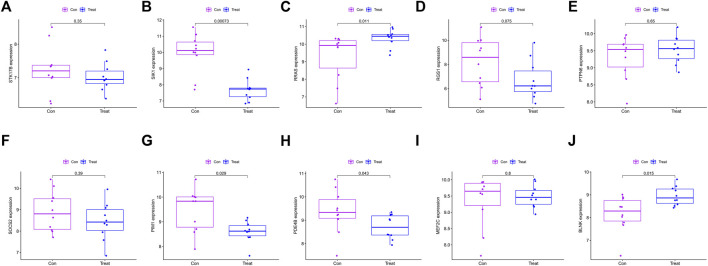
Box plot showing the difference in expression of hub genes in OA group **(A)** STK17B; **(B)** SIK1; **(C)** RRAS; **(D)** RGS1; **(E)** PTPN6; **(F)** SOCS2; **(G)** PIM1; **(H)** PDE4B; **(I)** MEF2C; **(J)** BLNK.

**FIGURE 7:**
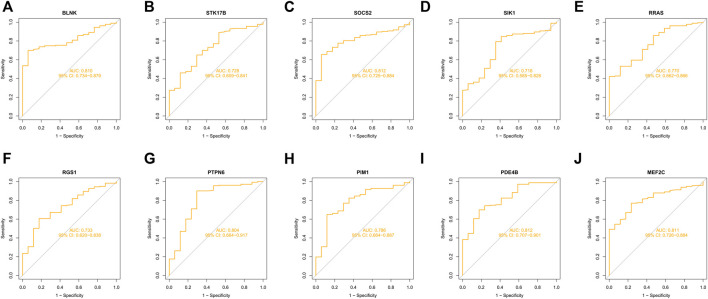
ROC curves of key genes for MDS diagnostics **(A)** BLNK; **(B)** STK17B; **(C)** SOCS2; **(D)** SIK1; **(E)** RRAS; **(F)** RGS1; **(G)** PTPN6; **(H)** PIM1; **(I)** PDE4B; **(J)** MEF2C.

10 hub genes were found in the dataset between the MDS patient group and healthy controls, where BLNK, SOCS2, SIK1, RGS1, STK17B, MEF2C, and PDE4B showed a downregulation trend in MDS, however, PIM1, RRAS, and PTPN6 showed opposite changes (Figures 5A–J). In OA we found that SIK1, PIM1, and PDE4B showed a downregulation trend, while RRAS and BLNK showed an upregulation trend (Figures 6A–J). Thus, we found that SIK1, PDE4B and RRAS are the genes that share the trend of both changes. These findings suggest that all these candidate genes have diagnostic potency and can be of diagnostic value for both diseases. To verify the value of hub genes in clinical applications, the AUCs of the 10 hub genes associated with MDS were therefore 81.00, 72.80, 81.20, 71.80, 77.00, 73.30, 80.40, 78.60, 81.20, and 81.10% (Figures 7A–J).

### Genetic variation

**FIGURE 8:**
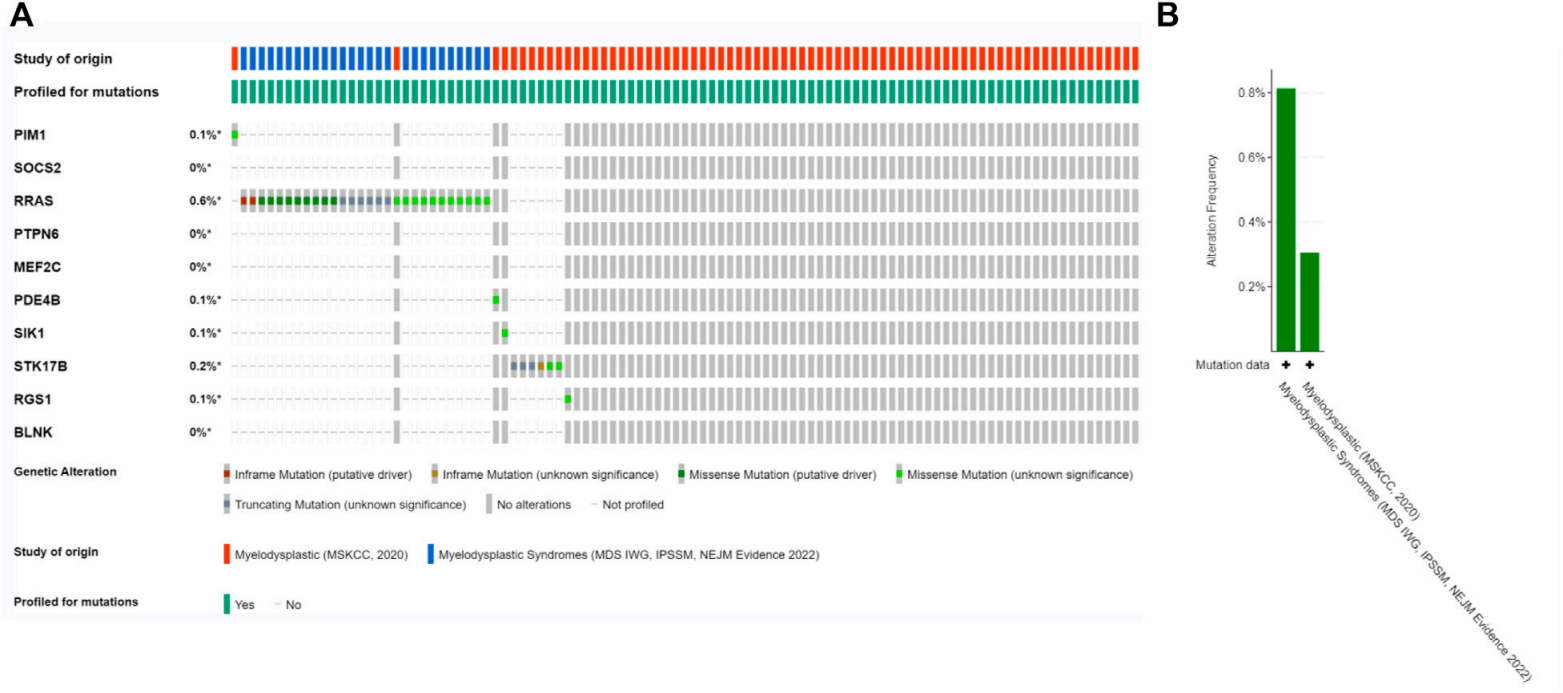
Genetic alterations. **(A)** Representation of genetic alterations showing genetic alterations in 10 key genes, which were altered in 756 (1%) of 7554 MDS patients; **(B)** illustrates the total frequency of alterations in 10 hub genes.

Since there is no dataset for OA disease to analyze gene mutations, in this section we will co-DEG the Hub gene in the MDS. The alteration status of the 10 Hub genes was analyzed using data from MDS patients from ecBioPortal Cancer Genomics Database. The frequency of alterations in each hub gene is shown in Figure 8A. The most alterations were found in RRAS, STK17B1 (0.6%, 0.2%, respectively), where missense mutations were the predominant type, and 756 (1%) of the other 7554 MDS patients (Figure 8B).

### Analysis of immune infiltrating cells

**FIGURE 9:**
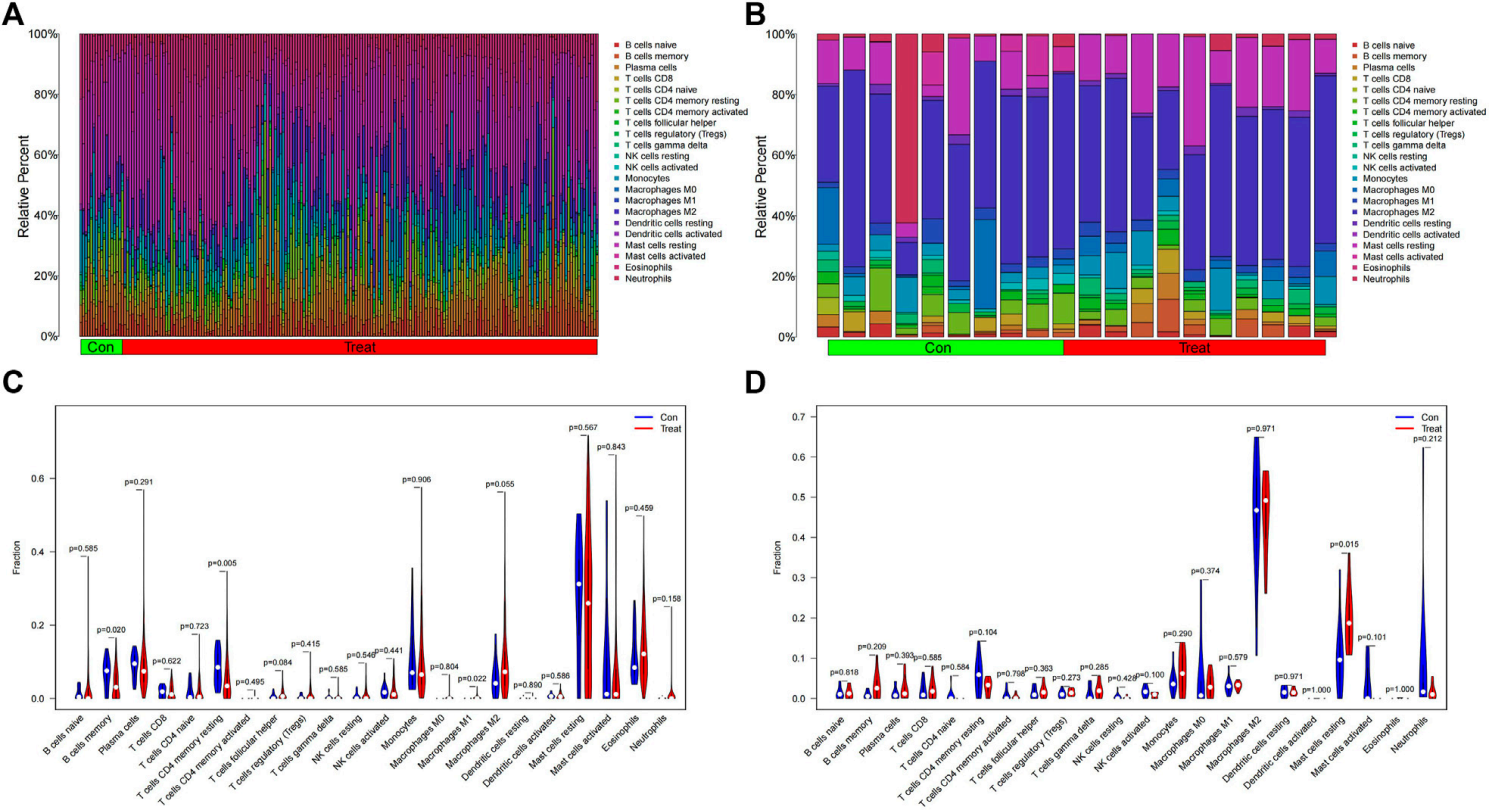
Immune cell infiltration analysis. **(A,B)** Relative number of immune cells; **(C,D)** Analysis of the difference in the relative number of immune cells.

For infiltrating immune cells, Figures 9A, B shows that the percentage of infiltration of 22 immune cells is further demonstrated in the immune cell ratio box plots. The analysis of the differences in immune cell infiltration in MDS and OA samples was visualized by violin plots, with *p* < 0.05 as a significant difference. The results showed that there were significantly more Macrophages M1 in the MDS group than in the normal group (*p* = 0.022), while the B cells memory (*p* = 0.020) normal group and T cells CD4 memory resting (*p* = 0.004) of the MDS group were significantly less than in the normal group (Figure 9C), while in the OA disease group Mast cells resting was significantly different from the normal group in terms of relative number and was highly expressed in the disease group (Figure 9D).

## Discussion

The relationship between MDS and autoimmune diseases is receiving increasing clinical attention, and MDS, like other malignancies, can present with various paraneoplastic immune phenomena such as vasculitis, arthritis, Sweet syndrome, and immune hemolytic anemia (Grignano et al., 2018; Fozza et al., 2022). Chondrocytes are an important factor in cartilage tissue to maintain articular cartilage homeostasis, and studies have confirmed that massive apoptosis of chondrocytes and abnormal expression of immune factors are one of the important causes of OA (Ishii et al., 2022). A total of two datasets were retrieved from GEO in this study, and 52 common genes in MDS and OA were obtained compared to normal controls. Enrichment analysis was performed to identify some GO terms and KEGG pathways, and 10 genes that were strongly associated with MDS and OA were selected in the PPI network construction. The results showed that the AUC values of the above genes were all greater than 0.7, suggesting their diagnostic ability with high reliability, and all genes, except PDE4B, had correlation with immune infiltrating cells.

We further performed PPI network analysis on 52 co-expressed genes, constructed the PPI network composed of proteins, and screened the 10 most critical hub genes in this network by analysis. B-cell ligand protein (BLNK) is a B-cell-specific bridging protein, and silencing BLNK can slow the progression of OA by regulating NF-κB signaling pathway (Cheng Y. et al., 2021). Bioinformatic analysis revealed that MDS patients with altered BLNK had poorer (Le 2019). Kurata et al. (2021) found that an inverse relationship between BLNK and C/EBPβ expression was also noted in pre-B-ALL cases, and that high levels of CEBPB expression were associated with shorter survival in pre-B-ALL patients with BLNK downregulation. MEF2C, a member of the MEF2 family, was originally identified in skeletal muscle cells and plays an important role in myoblast differentiation (Zeng et al., 2022). At the same time, MEF2C has been associated with processes such as differentiation and development of cardiac muscle, neural crest and chondrocytes (Zhang and Zhao, 2022). Studies have indicated that the p38 inhibitor Pamapimod protects chondrocyte hypertrophy by inhibiting the p38/MEF2C pathway (Zhang et al., 2020). Recent studies have shown that MEF2C is aberrantly expressed in tumor progression such as hepatocellular carcinoma (Chen et al., 2022) and leukemia (Cante-Barrett et al., 2022). Suppressor of cytokine signaling protein 2 (SOCS2) is an important negative regulator of the inflammatory signaling pathway JAK/STAT pathway that regulates cascade changes in the inflammatory response and is associated with cancer as well as neurological as well as immune-related diseases (Li et al., 2021a). It has been reported that SOCS2 suppresses the inflammatory response, reducing chondrocyte apoptosis, and inhibiting the progression of osteoarthritis (Zhang et al., 2021). In addition, enhanced GH signaling *via* SOCS2 deletion accelerated growth plate fusion (Samvelyan et al., 2022).

Non-receptor-type protein tyrosine phosphatase 6 (PTPN6), expressed mainly in hematopoietic stem cells, regulates receptor tyrosine kinases by binding to target proteins and dephosphorylating tyrosine substrates (Ahn et al., 2022; Luo et al., 2022). The m6A methyltransferase METTL14 promotes proliferation and osteogenic differentiation of bone marrow mesenchymal stem cells in steroid-associated femoral head necrosis by upregulating m6A levels of PTPN6 and activating the Wnt signaling pathway (Cheng C. et al., 2021). Similarly, proto-oncogene PIM1, a member of the PIM family of serine/threonine kinases, was identified from lymphoma samples as a frequently activated gene, which is highly conserved evolutionarily (Zhang H. et al., 2022). A recent study suggests that PIM1-quercetin docking may play an important role in the treatment of osteoarthritis (Ma et al., 2019). Phosphodiesterases (PDE) are specific enzymes for intracellular cAMP and cGMP degradation, and the PDE4 family is closely related to the regulation of inflammatory responses, with the PDFAB isoform playing an important role (Zhou et al., 2022). In PsA, dysregulated miR-23a expression promotes synovial inflammation by enhancing synovial fibroblast activation through PDE4B signaling (Wade et al., 2019). In addition, circPDE4B acts as a scaffold to promote RIC8A-MID1 binding, thereby reducing RIC8A-dependent activation of the P38 signaling pathway and thus regulating OA progression (Shen et al., 2021).

RRAS affects malignancies such as gliomas and pancreatic ductal adenocarcinomas (Xiao et al., 2019). Clinical studies point to the occurrence of pediatric myelodysplastic syndromes accompanied by germline RRAS mutations (Catts et al., 2021). RGS1, STK17B, and SIK1 have not yet been reported between OA and MDS disease.

In this study, we found that differences in immune-related or inflammation-related indicators differed in the two types of tumors and could be important indicators for differential diagnosis. Based on this, we compared the presence of immune-related differential genes among the differential genes in the two types of tumors and found that all nine genes play an important role, thus suggesting that there are differences in the immune environment in the two diseases and that such differences may provide a reference for future diagnosis and treatment. Due to the quantitative limitation problem of the OA dataset, only the common hub genes associated with MD were evaluated for diagnostic value in this study, and five genes (BLNK, MEF2C, SOCS2, PTPN6, and PDE4B) were found to have high diagnostic value and could be diagnostic biological markers for MDS. Differences in immune-related indicators exist in OA and MDS and can be important indicators for differential diagnosis. Based on this, we compared the presence of immune-related differential genes in OA and MDS and found that nine hub genes both play an important role, and such differences may provide a reference for future diagnosis and treatment.

This study elucidates the potential relationship between OA and MDS through bioinformatics methods, and provides a more reliable target for the in-depth study of the diagnosis and prediction of the two diseases. However, we further verify these target genes through *in vivo* and *in vitro* experiments to understand their specific functions. After the gene level is confirmed, we will explain the correlation between molecular mechanism level and clinical through clinical multi center or single center cohort research, It aims to provide a theoretical basis for clinical treatment and development of targeted drugs.

## Data Availability

The datasets presented in this study can be found in online repositories. The names of the repository/repositories and accession number(s) can be found in the article. For osteoarthritis, the GEO dataset name: GSE55235, Repository link: https://www.ncbi.nlm.nih.gov/geo/query/acc.cgi?acc=GSE55235. For myelodysplastic syndrome, the GEO dataset name: GSE19429, Repository link: https://www.ncbi.nlm.nih.gov/geo/query/acc.cgi?acc=GSE19429.
