# Supplementary figures and images for: Bioinformatics gene analysis of potential biomarkers and therapeutic targets of osteoarthritis associated myelodysplastic syndrome
Source: Front Genet. 2023 Mar 9;13:1040438. doi: 10.3389/fgene.2022.1040438 (PMC10034022; doi:10.3389/fgene.2022.1040438)

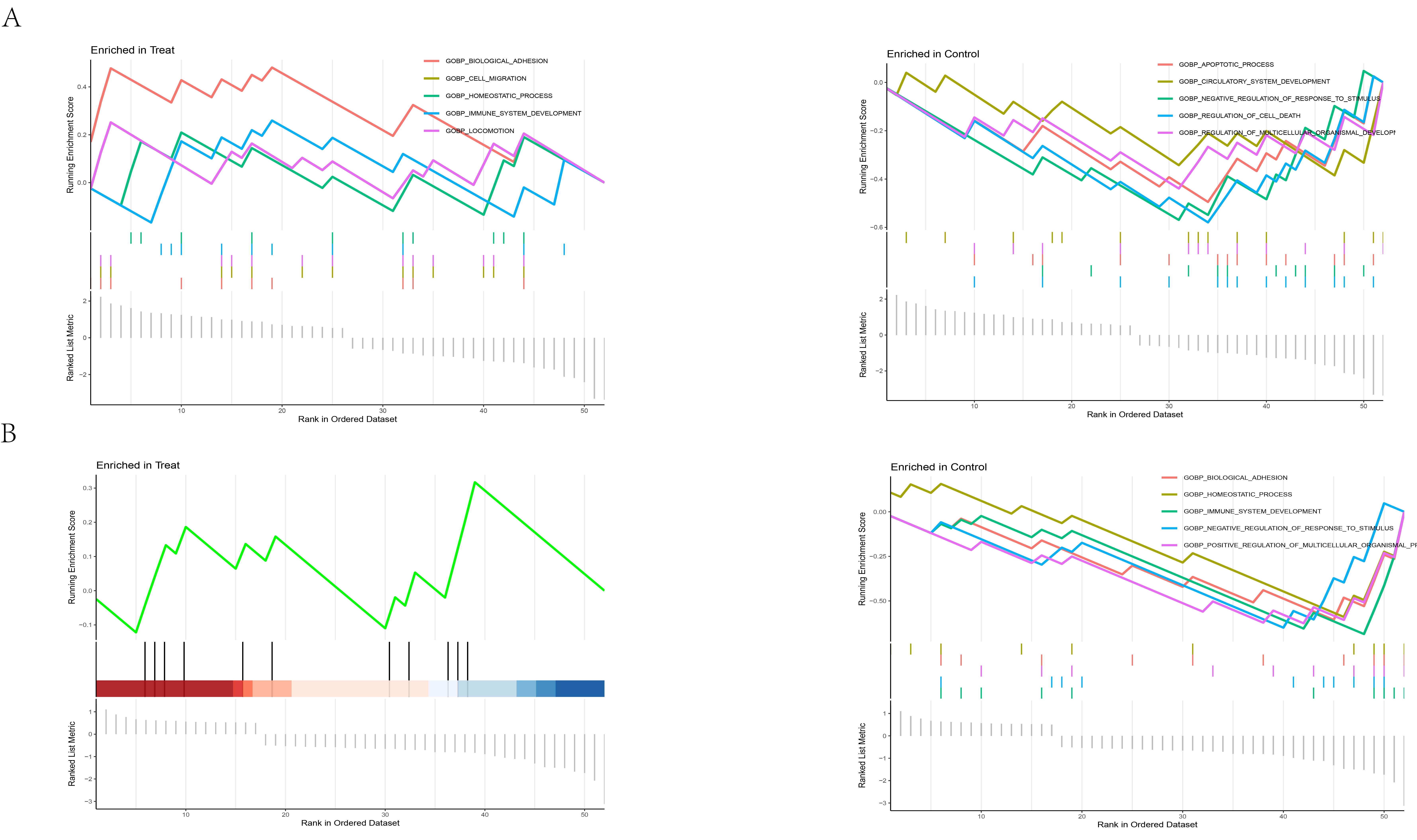

Supplement: Supplementary file 1 [file Image1.JPEG]
